# 

**Published:** 1887-06-25

**Authors:** 


### CONTENTS.

**Buttercups and Daisies** - - - - - - 209

**Words of Consolation.**—XXXVIII. The Work of the Holy Ghost - - - - - - 210

**Counterparts.** By a STUDENT OF EASTERN PHILOSOPHIES. Chap. III - - - - - 211

**The Doctor's Pulpit**: Rheumatism - - - - 213

**Hospital Sunday at the Synagogue** - - 214

**The National Pension Fund** - - - 214

**Mr. Spurgeon on Hospitals** - - - - 215

**Notes and News.**—Hospital Appointments.—Hospital Saturday Bazaar at Lincoln.—Hospital Collecting-Boxes in Wimbledon Camp.—The Ormsby Cottage Hospital.—The Supply of Goods to Hospitals.—The Paddington Children's Hospital.—Vacancies.—The Poplar Accident Hospital.—The Wigan Infirmary.—The City Orthopædic Hospital.—Hospital Collections at Worthing.—Proposed New Hospitals.—The Royal South Hants Infirmary.—Bazaars and Entertainments in Aid of Hospitals.—Annual Report of the Birmingham and Midland Skin Hospital.—The Edinburgh Association for Incurables.—The Sunderland Infirmary.—The Glasgow Hospitals, etc., etc. - - - - - 216

**Annotations.**—Hospitals "Sitting on the Fence."—A Happy Thought.—Provident Dispensaries - - 219

**After the Battle** - - - - - - 220

**Incidents of the Hospital Meetings.** By ONE WHO WENT TO THEM ALL - - - - - 221

**In- and Out-Patients' Hospital Diary** - 223

**The Children's Corner.**—Puzzles, Answers, etc. - vi

**Amusements with Profit** - - - - - vi

